# Spatial Normalization of Reverse Phase Protein Array Data

**DOI:** 10.1371/journal.pone.0097213

**Published:** 2014-12-12

**Authors:** Poorvi Kaushik, Evan J. Molinelli, Martin L. Miller, Weiqing Wang, Anil Korkut, Wenbin Liu, Zhenlin Ju, Yiling Lu, Gordon Mills, Chris Sander

**Affiliations:** 1 Computational Biology Center, Memorial Sloan-Kettering Cancer Center, New York, New York, United States of America; 2 Department of Systems Biology, The University of Texas M. D. Anderson Cancer Center, Houston, Texas, United States of America; 3 Division of Quantitative Sciences, The University of Texas M. D. Anderson Cancer Center, Houston, Texas, United States of America; Rice University, United States of America

## Abstract

Reverse phase protein arrays (RPPA) are an efficient, high-throughput, cost-effective method for the quantification of specific proteins in complex biological samples. The quality of RPPA data may be affected by various sources of error. One of these, spatial variation, is caused by uneven exposure of different parts of an RPPA slide to the reagents used in protein detection. We present a method for the determination and correction of systematic spatial variation in RPPA slides using positive control spots printed on each slide. The method uses a simple bi-linear interpolation technique to obtain a surface representing the spatial variation occurring across the dimensions of a slide. This surface is used to calculate correction factors that can normalize the relative protein concentrations of the samples on each slide. The adoption of the method results in increased agreement between technical and biological replicates of various tumor and cell-line derived samples. Further, in data from a study of the melanoma cell-line SKMEL-133, several slides that had previously been rejected because they had a coefficient of variation (CV) greater than 15%, are rescued by reduction of CV below this threshold in each case. The method is implemented in the R statistical programing language. It is compatible with MicroVigene and SuperCurve, packages commonly used in RPPA data analysis. The method is made available, along with suggestions for implementation, at http://bitbucket.org/rppa_preprocess/rppa_preprocess/src.

## Introduction

In the last decade, the study of cancer biology has been accelerated by many technological advances, enabling analyses of the genome at both high resolution and throughput. This has led to the identification of mutations and biomarkers specific to various cancer types and patient sub-groups. However, clinical trials of targeted therapy guided by these studies have met with less success [1], [2]. One of the reasons for this is that while the causes of cancer are genetic, they result in cellular malfunction at the level of proteins. While changes in each level may be observed discretely, they are related intimately through processes such as translation of mRNA to protein and the control of gene transcription by proteins. Further, proteins can interact with metabolites post-translationally. This increases the complexity of the proteome via the existence of multiple forms of – e.g. phosphorylated, nitrosylated and methylated – molecules that vary in function. There is hence a need for reliable and affordable methods for protein measurement, at a scale capable of complementing today's genomics studies, so that together, they may reveal the mechanisms driving cancer.

Reverse phase protein array (RPPA) technology is a powerful technique for measuring the activities of proteins from tissue- and cell-derived lysate. It is an inexpensive, high throughput, quantitative method with low sample requirements, making it ideal for large-scale proteomic profiling studies. In RPPA, small (∼µl) amounts of lysate extracted from biological samples under study are evenly spotted onto the surface of glass slides coated with an absorbent material such as nitrocellulose. A single RPPA slide of 2 cm×5 cm can be used to simultaneously measure the levels of a protein in thousands of samples at a time, using an automated and efficient procedure that can be scaled up to hundreds of proteins [3]–[5]. Each slide is probed with a primary antibody against the protein of interest, sensitive to pg-ng of protein [6], followed by a secondary antibody. A colorimetric or fluorescent signal is then generated, in proportion with the secondary antibody bound, and may be quantified to yield estimates of relative protein concentration in each sample.

RPPA design has several advantages over existing methods for protein detection. Unlike methods such as Western Blotting and 2D-Gel Electrophoresis, RPPA has high throughput and low sample requirements. While other assays such as multiplexed flow-cytometry and microsphere-based assays retain some of these advantages, they are far more expensive than RPPA and are often more labor intensive [7]. Mass spectroscopy (MS), which is another method used in large-scale protein level studies, can analyze the proteins in a sample using both unbiased and targeted approaches. However, current methods for MS require high sample volumes and the time required for sample analysis can be high. Reverse Phase Protein Arrays have enabled studies of protein networks implicated in different cancers [8], [9], infectious disease [10] and the responses of cells to various drugs [11]–[13]. However, many of the factors that make RPPA an appropriate choice for proteomics studies also introduce noise into the data. For example, the use of targeted antibodies enables the measurement of low-abundance proteins, but low antibody specificity can lead to promiscuous binding and false positives [14], [15]. Similarly, the handling of low sample volumes can lower the signal to noise ratio of the results [16]. The reliability and reproducibility of RPPA data are a key determinant of the utility of such studies. We examine one factor that contributes to noise in the RPPA data – spatial heterogeneity – and describe a method for correcting it, thereby enhancing the quality of the data.

**Figure 1:**
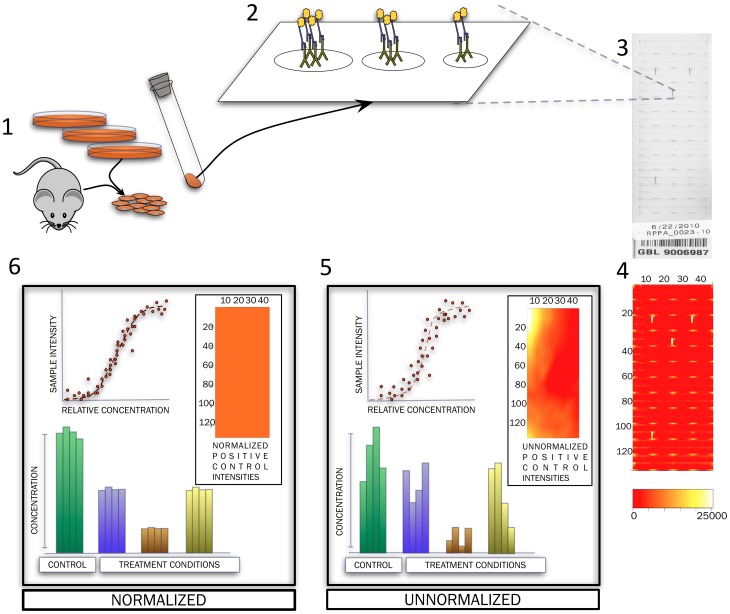
Steps in the acquisition and processing of RPPA data. Cells derived from different in vitro and in vivo systems are lysed and protein extracted (1). Serially diluted extracts are printed onto the surface of slides (2) where primary and secondary antibodies bind to the protein of interest and generate a signal proportionate to the amount of protein in each sample. Each slide can accommodate 5808 printed spots, for different numbers of total samples depending on the layout and number of dilutions used (3). Readouts obtained are translated to sample intensities after scanning and processing of the slides (4). Intensities of positive control spots (horizontal yellow spots in (4)), which are technical replicates of each other, may be used to evaluate and correct spatial variation observed in each slide. Spatial correction of data can improve data quality resulting in better estimates of relative protein concentration and improved agreement between inter- and intra-slide replicates from various experiments.

Spatial variation in RPPA slides occurs due to unequal exposure of the slides to the experimental reagents used. This causes non-uniform signal generation, resulting in systematic variations across the area of each slide. Spatial heterogeneity is obvious when identical samples distributed over a slide produce variable signal intensities. Consequently, variance across identical samples serves as a reference with which one can measure and then correct errors arising from this heterogeneity (Fig. 1). We show that spatial differences affect the results of RPPA data obtained from diverse biological datasets. We use a simple, flexible and powerful 2D interpolation method to normalize the data, resulting in significantly enhanced data quality as measured by improvements in reproducibility and the signal to noise ratio of the results. Also, data from antibodies that were previously unusable are rescued with the method, improving the utility of the studies performed. R code for the method is provided as a package that can be used in conjunction with MicroVigene, currently a widely used platform for the analysis of RPPA data.

## Materials and Methods

### Data sets analyzed using normalization routine

RPPA data for this study were obtained from slides printed with various human cell-line and tumor derived samples and probed with antibodies specific to proteins relevant to the study. The details of the method are provided in the results. We tested this method on the following data sets.

#### 1) Set A - Quality control samples

This dataset was comprised of 16 slides, each identically printed with sample and then queried with a single primary antibody. The samples in these slides were obtained from a quality control study performed in the M.D. Anderson Cancer Center RPPA core-facility and a list of the antibodies used is provided in table S2.

#### 2) Set B - Human melanoma cell line-derived samples

This data set was obtained from experiments performed in-house in the Sloan Kettering Institute. The melanoma cell line SKMEL-133, a ^V600E^BRAF/PTEN null mutant cell line kindly gifted to us by Dr. David Solit, MSKCC [17], was perturbed with 10 small molecule inhibitors (table S1) targeting specific kinases that control cell death and proliferation. Cells were treated with each drug individually as well as with all pairwise combinations of the drugs. Three biological replicates of each experimental condition were generated, constituting approximately 300 samples that were measured with RPPA. Cell lysate from each sample was spotted onto slides and probed using 159 antibodies (table S2) to measure the quantities of clinically relevant proteins or phospho-proteins in those samples. Several of the slides were probed with the same antibody 2–3 times, resulting in a total of 238 slides and 53 antibodies with replicate slides.

#### 3) Set C – Miscellaneous anonymized samples

A data set comprised of 30 slides from cell-line data processed at the M.D. Anderson Cancer Center.

### Preparation, layout, printing and quantification of lysate array samples

**Figure 2:**
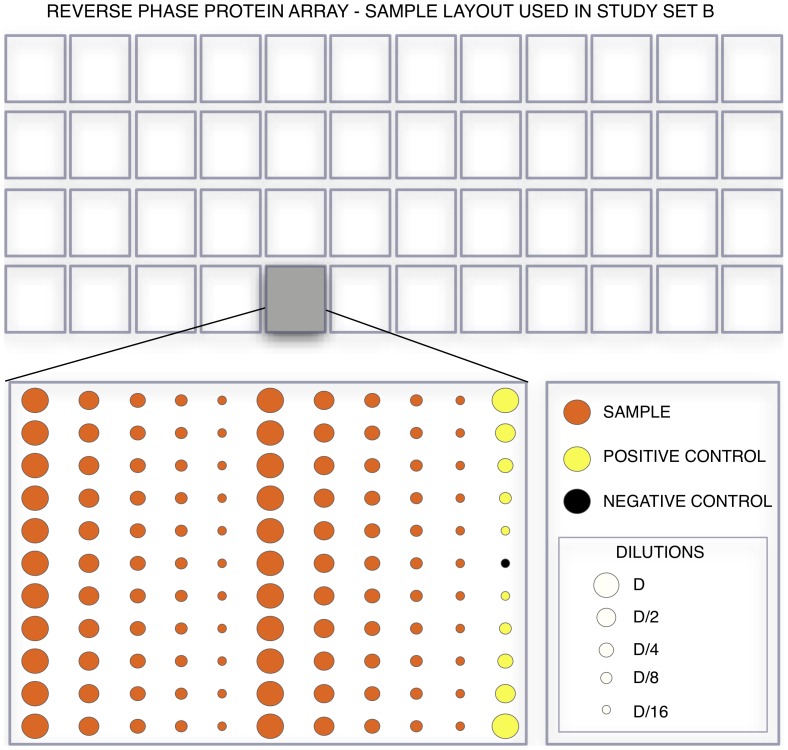
In the experimental design we use for the analysis of the samples in sets A and B, lysate is spotted in 96 arrays consisting of 22 samples, two positive controls and one buffer spot each. Each of the samples and the positive controls is printed in five 1∶2 serial dilutions each.

Homogenized cell pellets consisting of cellular proteins are derived from cells grown *in-vitro* or from tissue samples *in-vivo*. Samples are lysed and the protein extract obtained is diluted based on the design of each experiment. In the slides comprising the data sets in this study, each sample undergoes a ½ serial dilution four times, leading to a total of 5 concentrations per sample. These initial serial dilutions are performed manually. Diluted samples are then robotically spotted onto the surface of slides coated with nitrocellulose. In our experimental design, each sample and positive control is printed in five dilutions. The slides are laid out as grids of 132×44 spots, comprised of 48 subgrids containing 121 spots each. Thus, each subgrid accommodates 22 samples and 2 positive control samples, in 5 dilutions each. A subgrid is also printed with a single buffer spot that serves as a negative or background control. Each slide thus accommodates 1056 serially diluted samples and 96 positive control samples (with 5 dilutions per sample), and an additional 48 negative control spots (Fig. 2). The positive control spots, are printed at fixed intervals across the length and breadth of each slide, and are technical replicates of each other, obtained from a single batch of standard mixed cell lysate [18]. Since the controls are designed to contain sufficient amount of each of the proteins in the antibody panel for reliable detection, similar levels of the concerned protein should also be detected in experimental samples when the appropriate dilution of antibody is used. The negative control spots consist of buffer containing no protein and are hence informative of the level of background signal generated.

Protein in each sample is quantified by washing the slide with a solution of primary antibody followed by secondary antibody. The biotinylated secondary antibody interacts with a streptavidin bound peroxidase to catalyze the deposition of a biotinylated brown tyramide compound on the surface of the spot. The intensity of the colored signal thus generated is proportional to the amount of secondary antibody and protein bound to the slide. Signal intensities obtained by scanning images of the slides were quantified by MicroVigene software [19]. These are then translated into relative protein concentrations using an R package called SuperCurve [20]. SuperCurve estimates the concentrations of all the samples on a slide with respect to one another. The estimation is based on the assumption that all the samples on a slide lie on a single dose response curve, since the hybridization kinetics of all samples have similar chemistry. The curve thus obtained may be used to obtain the relative concentration of each sample on the slide.

### Assessment of data quality

The effectiveness of normalization was assessed based on the behavior of biological and technical replicates compared before and after normalization. Successful normalization should reduce noise, resulting in improved comparability of data and should bring replicates closer to each other. We define **technical replicates** as spots that are printed from lysate that was obtained from a single batch of cells in a single experiment. When printed onto a single slide, they are called intraslide replicates and when printed onto different slides, they are interslide replicates. For example, all the positive control spots belonging to a single dilution on a single slide are intraslide technical replicates because they were obtained from a single mix of cells and subjected to dilution in a batch before the lysate was printed onto slides. **Biological replicates** are spots that are printed from cell lysate obtained from cells that were subjected to the same experimental conditions, but in separate batches. For example, in procuring dataset B, SKMEL-133 cells were grown in 3 different petri-dishes, and each was subjected to normal medium spiked with a dose of EGF ligand. They were then used to yield three separate cell pellets that when lysed and printed onto a slide, gave rise to biological replicate spots.

We expect technical and biological replicates to have different degrees of variability. Similarity of technical replicates is indicative of the reliability and uniformity of steps in the procedure such as printing, probing and scanning. On the other hand, biological replicates may vary for a number of reasons. The heterogeneity inherent to populations of cells obtained from both cell lines and tumors may make subsets of such populations behave differently when subjected to the same treatment. Several other factors could introduce biological variation, such as time to freezing and the presence of stromal and endothelial cells in tumor-derived samples, or the sample preparation method used [21]–[24]. Thus when technical variability is low, the differences between biological replicates can yield useful information about cellular variability in the samples studied.

To determine how spatial normalization improves the quality of RPPA data, we calculated

1. Agreement between interslide and intraslide technical replicates across 16 pairs of duplicate slides from dataset A, and 53 pairs of duplicate slides from dataset B.
2. Agreement between intra-slide biological replicates in a 238-slide melanoma cell line study.

Agreement was evaluated with the Pearson's correlation (ρ) between corresponding spot intensities (I_A_ and I_B_) across duplicate slides and the coefficient of variation (%CV) between replicates within-slide, where μ denotes the mean and σ the standard deviation of the spot intensities (I) or protein concentrations (P) measured. 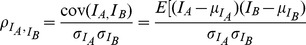
(1)

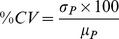
(2)


## Results

### Bilinear interpolation of correction factors to remove spatial biases in RPPA data

The central assumption is that in the absence of spatial variance all positive controls of a given dilution should yield equal intensities. Consequently, observed variability of positive control intensities is a survey of the spatial bias on the slide. With this information, we can systematically factor out the spatial bias at any location based on neighboring positive control intensities.

We define the relationship between the measured sample intensity I(x,y) and the true intensity I′(x,y) in terms of a correction factor CF(x,y) that represents spatial variance.
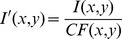



Correction factors are simply the ratio of positive control intensities PCI(x,y) to some reference intensity <PCI>. 
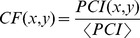



Here, we choose the mean positive control intensity <PCI> to be the reference intensity. CF values above 1 indicate regions on the slide where there is a bias towards larger intensities. CF values below 1 indicate regions on the slide where there is a spatial bias towards smaller intensities.

However, these correction factors are not directly calculable at sample locations precisely because those locations do not contain positive controls. To compensate for this missing information, we use interpolation to approximate *pseudo*-positive control intensities at the sample locations.

Interpolation is the calculation to approximate the value of a function f(x,y) at specific locations (x,y) given fixed knots or measured function values at neighboring locations f(xc, yc) and is analogous to “Connect the Dots”. Linear interpolation means we connect the dots with lines. The points lying on the lines between the dots are the interpolated values, and the dots themselves are fixed knots or anchor points. The interpolated values are approximations inferred based on nearest neighbor data. In this case, we will use the measured positive control intensities to interpolate or approximate pseudo-positive control intensities at all locations on the slide.

Consider a location (x,y) that lies between four measured positive control spots with corresponding intensities *PCI(x_a_,y_a_), PCI(x_a_,y_b_), PCI(x_b_,y_a_), PC(x_b_,y_b_)*.
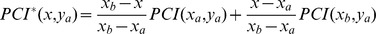


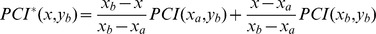


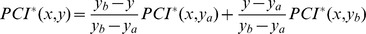



These are pseudo-positive control intensities (indicated by an asterisk) in that they are approximations for what a control intensity at that location would have been had it been spotted with control sample. The correction factors at these locations are calculable with simple division by the reference positive control intensity.
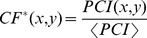



The bilinear interpolation calculation described above reflects only our assumptions about the smoothness of the spatial bias between measured positive control locations. It says nothing about the relationship any sample intensity has to another sample intensity. A similar correction can be applied after performing a cubic spline interpolation between the correction factors. Overall, the results of normalization using spline interpolation are similar to those with bilinear interpolation (table S3). Hence we use the simpler of the two, bilinear interpolation, for normalization (Figure S1). Further, in the sample and control format used in our experiments, there are 96 sets of positive controls printed in 5 dilutions each. We use the median of each set as anchors for our interpolation step as this dilution is the most likely to be in the linear range of the assay for the set of antibodies used in the experiment. Users of the method are encouraged to design their experiments such that all the query samples are contained within the interpolation region of the positive controls. In our design, a portion of the slide (1/12^th^) does not have positive controls at its periphery and hence, each sample in this region was normalized by the closest correction factor evaluated.

### Spatial normalization improves Coefficient of Variation between biological replicates

**Figure 3:**
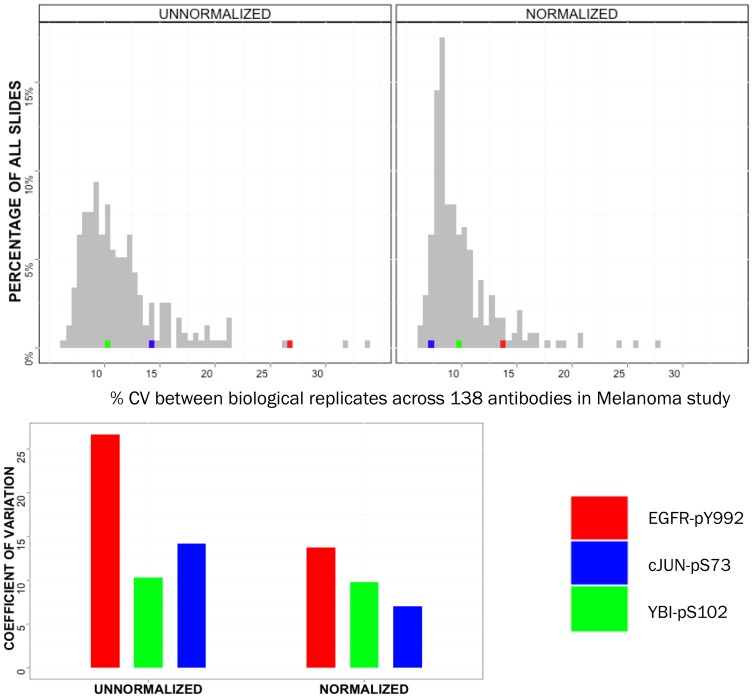
Coefficient of variation (%CV) of biological replicates across all antibodies before and after normalization clearly improve with normalization. The degree of improvement varies from antibody to antibody (higher for EGFR-pY992 and cJUN-pS73 than YB1-pS102) and is significant for many antibodies relevant to signaling in the melanoma cell lines studied.

Spatial normalization improves agreement between intraslide biological replicates in dataset B and ‘rescues’ previously discarded slides enabling further analysis of these proteins. Melanoma cell line samples were acquired for a large study aimed at understanding the basis of RAF inhibitor resistance in certain melanoma cell lines. Cell lysate was obtained from a melanoma cell line SKMEL-133 and subjected to various drug treatment conditions in triplicate, resulting in approximately 300 samples that were then quantified using RPPA. Agreement between the biological replicates was calculated before and after normalization. Around 10% of the slides (25/238) show increases of over 5% in agreement between biological replicates after normalization whereas only 1.2% (3/238) slides show a worsening of CV by over 5% with normalization. Despite increased agreement overall, biological replicates show different degrees of improvement with spatial normalization (Fig. 3).

**Figure 4:**
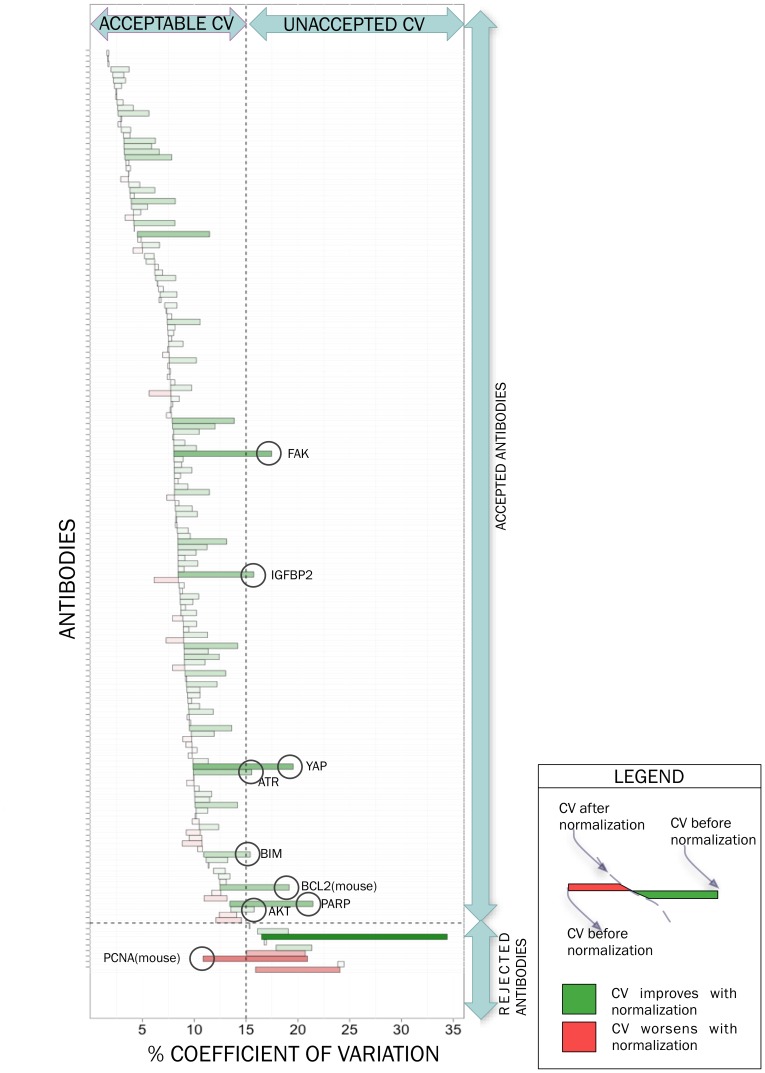
Spatial normalization reduces variance between biological replicates in the majority of the slides comprising a melanoma cell line study. In the study, a cutoff coefficient of variation (CV) of 15% is used to decide whether slides are retained for biological analysis. After spatial normalization, CVs in 8 slides (Caspase 9, IGFBP2, ATR, COX2, FAK_pY397, BCL2(mouse), PARP, AKT) that were previously unusable drop to acceptable values. One slide - PCNA(mouse) - that had earlier been used in analysis is rejected after normalization.

The data from this study were used to train a mathematical model of melanoma biology in SKMEL-133. To maximize model accuracy, only data points with sufficient reliability were kept for model incorporation and training. Slides were selected if the average coefficient of variation (%CV) of biological replicates within each slide was seen to be less than or equal to 15%. This threshold was arbitrarily selected by the authors and is left to the discretion of the user. %CV, which is the ratio of the standard deviation between observations to the mean of those observations, expressed as a percentage, is a good measure of signal to noise in biological data and rises with noise in the data. A set of 168 slides was originally selected after discarding saturated and defective slides. Of the 168, when we evaluated %CV across all biological replicates in each slide, 15 slides were unusable because of %CV greater than 15%. After normalization, only 7 slides had %CV greater than 15%. The slides that were rescued by spatial normalization measured AKT, PARP, BCL2, BIM, ATR, YAP, IGFBP and FAK (Fig. 4). In certain cases, %CV appears to rise after normalization. This could reflect real noise present in the data. However, the cases where this occurs are those where %CV is significantly below the cutoff of 15% and hence this did not affect the selection of antibodies in our study. To further verify this result, we also calculated the Z′-factor [25] of each slide before and after spatial normalization. In agreement with the %CV improvements we observed in biological replicates, the per-slide Z′-factor evaluated in dataset B also improves in >98% of the slides used in the experiment (details and calculations provided in Fig. S3).

### Spatial normalization modestly improves the agreement between inter-slide replicates

To evaluate whether spatial normalization improved data quality significantly, we compared the agreement between technical and biological replicates before and after normalization. We compared the Pearson's correlation of the estimated concentrations of samples printed at equivalent locations across 69 pairs of duplicate slides procured independently from sets A and B to assess interslide reproducibility. Here, duplicate slides are slides that were printed with the same samples in equivalent locations on each slide.

**Figure 5:**
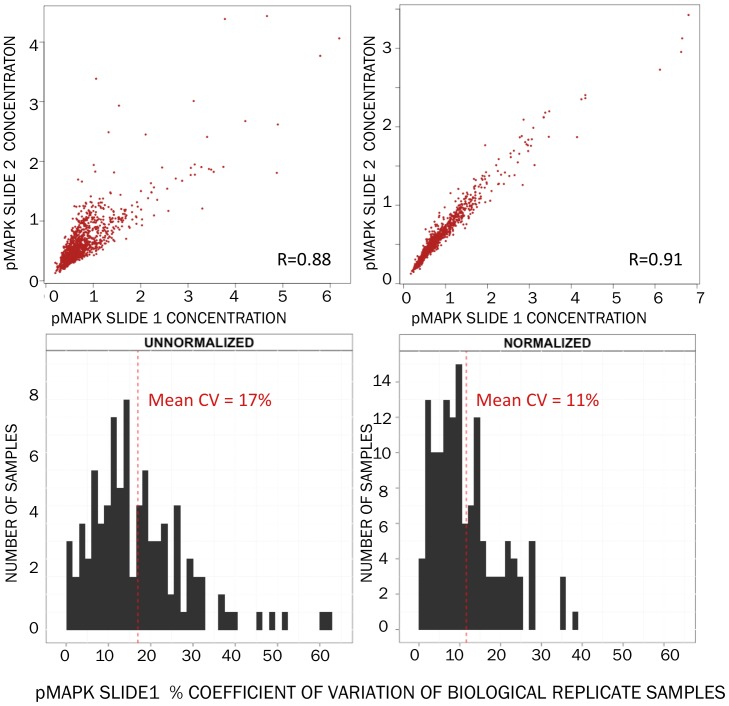
Correlation between concentrations of samples printed across duplicate slides increases slightly with normalization (upper panels, L→R, melanoma samples and probed with anti-pMAPK antibody). Coefficient of variation between the concentrations of biological replicates printed on one of these slides improves after normalization (lower panels, L→R).

Many slide pairs improve in overall correlation between concentrations, with only a minority of the slide pairs showing a large such improvement. Further, slides showing a modest improvement in the behavior of interslide technical replicates with normalization often show greater improvements in concordance of biological replicates (Fig. 5 and table S4). Earlier studies using RPPA have consistently shown that such correlations evaluated between the concentrations of interslide replicates are generally high [18] but may not be the best measure of improvement in data quality after normalization.

### Spatial normalization improves Intra-slide reproducibility of technical replicates

**Figure 6:**
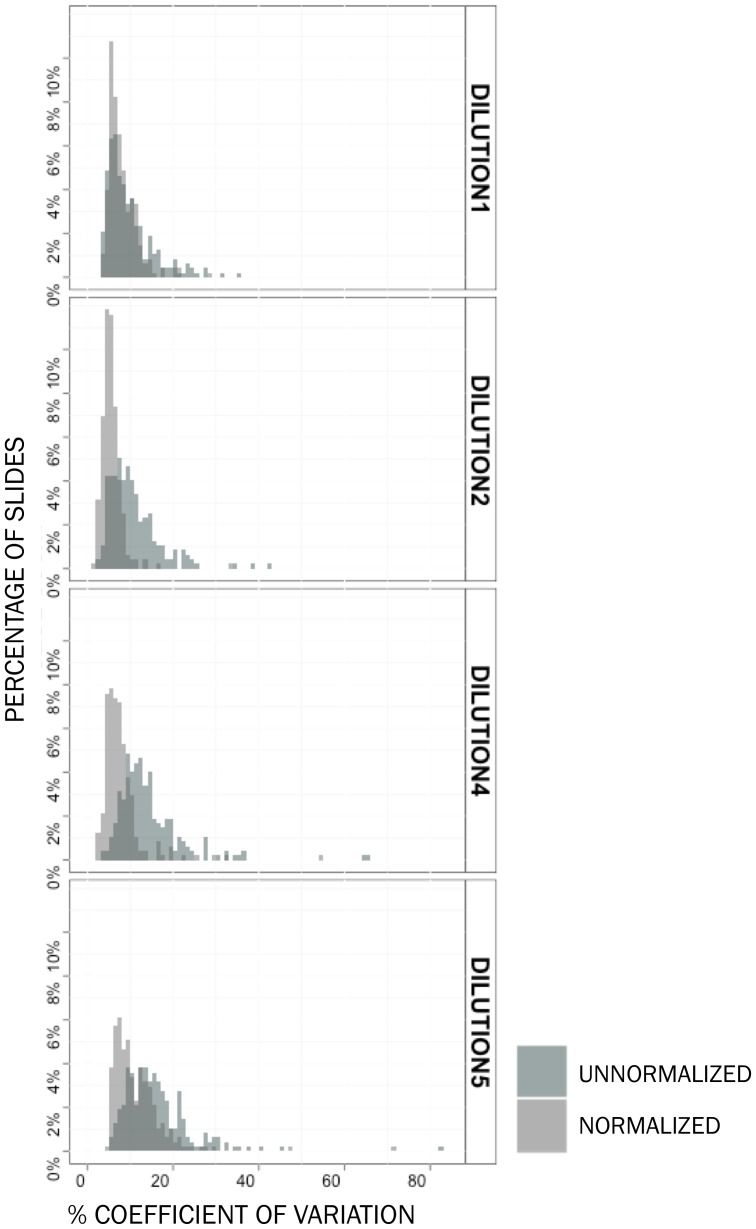
Coefficient of variation between intensities of intraslide technical replicates in dataset B decreases significantly with normalization. One out of 5 dilutions of positive controls is used for spatial normalization. The correlation of the remaining positive controls, which are technical replicates within each dilution, is observed after normalization. Correlations increase with normalization for each of the observed dilutions.

The slides evaluated for interslide reproducibility each have 480 positive controls, spotted as 96 sets of 5 dilutions each. The 96 points within a dilution are hence all technical replicates of one another. While the normalization method uses one of these sets, the median set, as anchor points for evaluating spatial variation and correction factors, we can use the remaining dilutions of the positive controls to measure %CV between each set before and after normalization. Doing this showed significant improvements in agreement between each such set of technical replicates, across most antibodies used. (Fig. 6) In the melanoma data-set, agreement between technical replicates showed an average improvement of 4%, with %CV falling from 12% to 8%, after normalization across slides probed with different antibodies. Further, 16 out of the 168 antibodies showed improvements of 10% or above in the coefficient of variation between technical replicates.

## Discussion

RPPA is one of two main techniques used in large-scale proteomics studies today – array based techniques and mass spectrometry. High-throughput, low sample requirement and high sensitivity make it a promising technology with which to examine protein networks in a variety of systems including cell lines and tissue samples. However, some of the features that make RPPA an appropriate choice for several kinds of proteomics studies, such as antibody-based detection, where antibodies have may different target-affinities and variable specificities, also add noise to the data it generates. Hence noise reduction and data normalization are essential for the successful application of RPPA. Our normalization technique evaluates one source of noise in RPPA data – spatial variation – and uses the measured variation to correct the data leading to increased reproducibility between duplicates in various studies. The method also makes the data from previously discarded, noisy slides usable in analysis, potentially expanding the scope of the biological questions that a set of RPPA experiments may address.

Among the genomics platforms, such as DNA microarrays, standards for experimental design and analysis have greatly improved the quality of those data and the scope of the studies that they enable [26]–[29]. This has lead to collaborative efforts such as the TCGA that have significantly enhanced our understanding of various cancers [30]. Among the protein activity measurement platforms, there are fewer methods that similarly address data quality. One such method [31], in which control samples are used to normalize for spatial and scaling errors in RPPA data successfully reduces intra-array replicate CV by up to 70%. However these improvements were the result of printing of as many control samples as each slide contained query samples and is hence expensive. Further, the published method was only applicable to a specified sample layout. Our method corrects a significant and systematic source of bias in RPPA data effectively reducing error in sample sets normalized with relatively few controls. Among the melanoma data we corrected, for instance, fewer than 2% of the samples were used to normalize a total of 5808 samples. Further, the method is flexible, allowing the user to correct for spatial biases in a variety of formats containing identical control samples that contain a level of the protein of interest that is within the linear detection range of the assay used. Others in the research community have similar goals and improved standardization of analysis methods will help realize the potential of RPPA in, e.g., characterizing the signaling response to drug treatment or in training mathematical models of biological systems.

As this manuscript was completed, two other alternative methods for spatial normalization of RPPA data were published [32], [33]. The first, by Troncale et al., uses a non-parametric model that takes into account every sample's Row and Column location while fitting the obtained intensities to relative protein expressions, thus adjusting for spatial effects along with other sources of variation addressed by the paper, such as background and total protein deposited at each spot. The method of Neeley et al. is similar in ideology to ours, in that it uses the variation observed between identical controls printed at various locations on each array to normalize for spatial effects. The correction is model based, and is specific to an array format that is commonly used in the community. While a systematic comparison of existing methods would help a user to select the method best suited to their experiment and data, this is beyond the scope of our current work. We compare the changes in reproducibility of data observed using our method with Neeley et al. across the antibodies in the melanoma dataset. These results are provided in (Figure S2). More extensive comparisons of the existing methods may aid in the selection of a set of standard methods for data normalization, or an improved understanding of what quantification and normalization methods work the best for different types of experiments. This would be beneficial to the RPPA community, where comparisons of experimental results are currently confounded by a lack of standardization.

**Figure 7:**
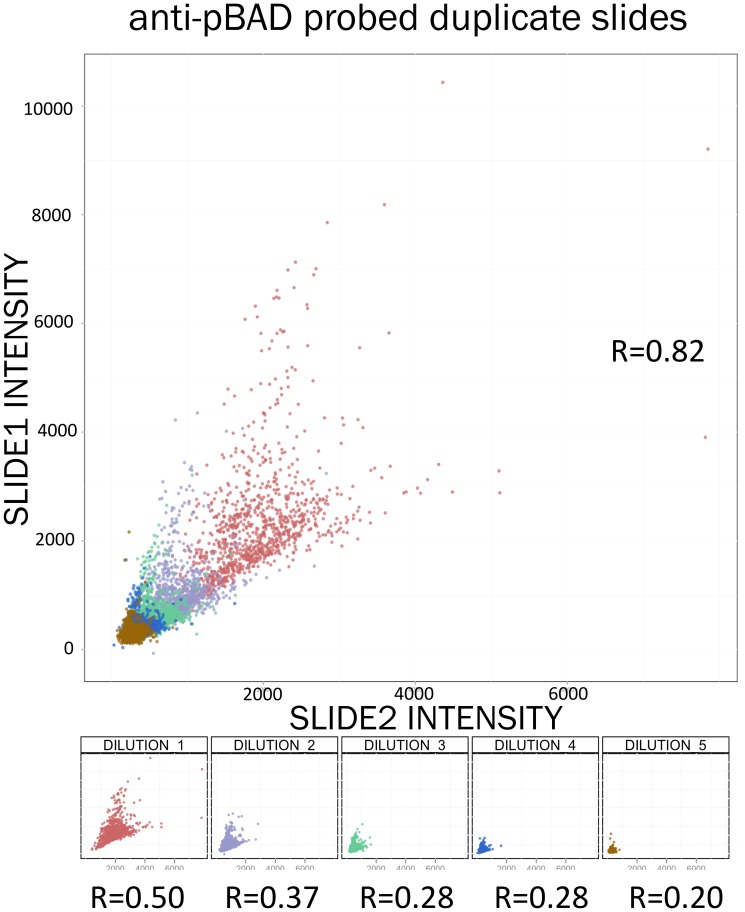
Correlation calculations performed using intensities of all spots printed onto duplicate slides may be a misleading measure of reproducibility because of experimental design that uses multiple dilutions to evaluate sample concentrations. In the case of two identical slides probed with anti-pBAD antibody, overall correlation coefficient R = 0.82 whereas correlations of the individual dilutions are lower.

A metric frequently used to assess data quality in RPPA is interslide and intraslide correlation between spot intensities of technical replicate spots [18]. While this gives us some confidence about the reliability of the results, it may not be an adequate measure of reproducibility. Since RPPA has a low dynamic range as compared to some other proteomics methods, this range is often expanded by printing multiple dilutions of each sample on the surface of a single slide. The dilutions of a sample may be widely separated in intensity, and correlations measured across all spot intensities on a slide may be biased by the range of intensities spanned by each slide (Fig. 7). When evaluating interslide correlations, we attempt to reduce this bias by comparing relative protein concentrations rather than intensities. Nonetheless, measures of intraslide technical and biological replicate equality can be more informative of data quality than Pearson's correlation. Other metrics of data quality, such as the Z′factor [25] and a Welch's t-statistic [34] to evaluate the mean difference between the positive and negative controls before and after normalization also showed improvements from normalization for the vast majority of samples. (figures S3 and S4).

The spatial normalization technique we implemented not only significantly decreased coefficient of variation improved agreement between biological and technical replicates within slides, but also made it possible to analyze the data from many slides that were previously unusable because of high variation. A particular example is our use of the antibody for PARP-1 in a study of melanoma samples subjected to various treatment conditions, where the %CV between biological replicates decreased from 21% to 13%, enabling more reliable use in the study after normalization. Poly (ADP ribose) polymerase (PARP) proteins (PARP-1 and PARP-2) play a critical role in controlling necrosis and apoptotic cell death. These PARP proteins are located inside the nucleus and take part in DNA-repair in response to DNA breaks and facilitate transcription, replication and DNA base excision repair [35]. PARP inhibitors (Olaporib, iniparib and veliparib) are undergoing clinical trials in BRCA mutated ovarian and breast cancer patients [36]. Furthermore, PARP-1 has been linked to altered control of p53-mediated DNA response and NFKappa-B response [37]. Consequently, accurate quantification of cleaved PARP-1 could be critical in understanding the complex signaling mechanisms involving PARP-inhibition as well as perturbations involving BRCA1 and BRCA2.

Other proteins similarly rescued in this and other studies could expand the scope of the biological problems addressed by RPPA. One context in which spatial normalization could be very relevant is in the analysis of tumor samples using RPPA, that due to requirements of throughput, cost and limited availability of patient material, are often unable to have sample replicates within slides. One such effort, belonging to the umbrella of TCGA projects, measures and compares protein abundance data across various tumors. In cases such as this, spatial variation alone could cause the appearance of differences that may bias the results. Hence it is very important that these data be appropriately normalized before use and analysis in other projects R code for our spatial normalization method can be used in conjunction with MicroVigene and SuperCurve. It is flexible and may be adapted to several different kinds of experimental designs, with the user specifying the locations of positive controls or other identical samples to be used as reference points for normalization.

Our method is one of several early efforts for the standardization and quality control of RPPA data. As data acquisition methods improve and RPPA moves into more widespread use, we advocate the adoption of common standards for the evaluation and correction, where possible, of systematic errors in RPPA data as well as in the analysis of these data to enable larger, multi-center studies and improve comparability across individual studies.

## Supporting Information

Figure S1 **Coefficient of variation between all biological replicates, and across 237 antibody slides used in a melanoma study, before and after normalization of sample intensities using bilinear interpolation and cubic spline.** Both methods result in greater agreement between replicates due to normalization.
(TIFF)

Figure S2 **Coefficient of variation between biological replicates in the melanoma study (SET B) appears to worsen for many antibodies when normalization is implemented using the method of Neeley et al.**
(TIFF)

Figure S3 **Spatial normalization improves the quality of the data from almost all the antibodies in a set of slides (Set B) printed with lysate from the melanoma cell line SKMEL-133. 30% of the slides which had a Z′-Factor of lower than 0.5 show Z′>0.5 after normalization.** Further, unusable data from nearly 11% of the slides (26/238) show a Z′>0.25 after normalization.
(TIFF)

Figure S4 **Spatial normalization increases the observed differences between the positive and negative controls in a set of slides (Set B).** 229 out of 238 slides (96%) of this set show a clearer separation between the controls after normalization.
(TIFF)

Table S1 **A list of the drugs used to perturb a melanoma cell line and the doses used, both singly and in all pairwise combinations.**
(XLSX)

Table S2 **All slides in the melanoma study with antibodies and dilution used in each.**
(XLSX)

Table S3 **Contains results of a comparison of duplicate slides obtained form studies conducted in SKI and MDA.** Results of this analysis are reported as interslide and intraslide replicate CVs in the results section of the paper.
(XLSX)

Table S4 **Compares the % CV between the concentrations of biological replicates printed from a melanoma cell line study.** CVs reported correspond to that before normalization, and to that after normalization with two methods – bilinear interpolation and cubic spline interpolation.
(XLSX)

Code S1 **Contains the R code for the method along with example data and guidelines for use.**
(ZIP)

File S1 **Contains details of supplementary performance assessment of the method.**
(DOCX)
