# Supplementary figures and images for: Spatial Normalization of Reverse Phase Protein Array Data
Source: PLoS One. 2014 Dec 12;9(12):e97213. doi: 10.1371/journal.pone.0097213 (PMC4264691; doi:10.1371/journal.pone.0097213)

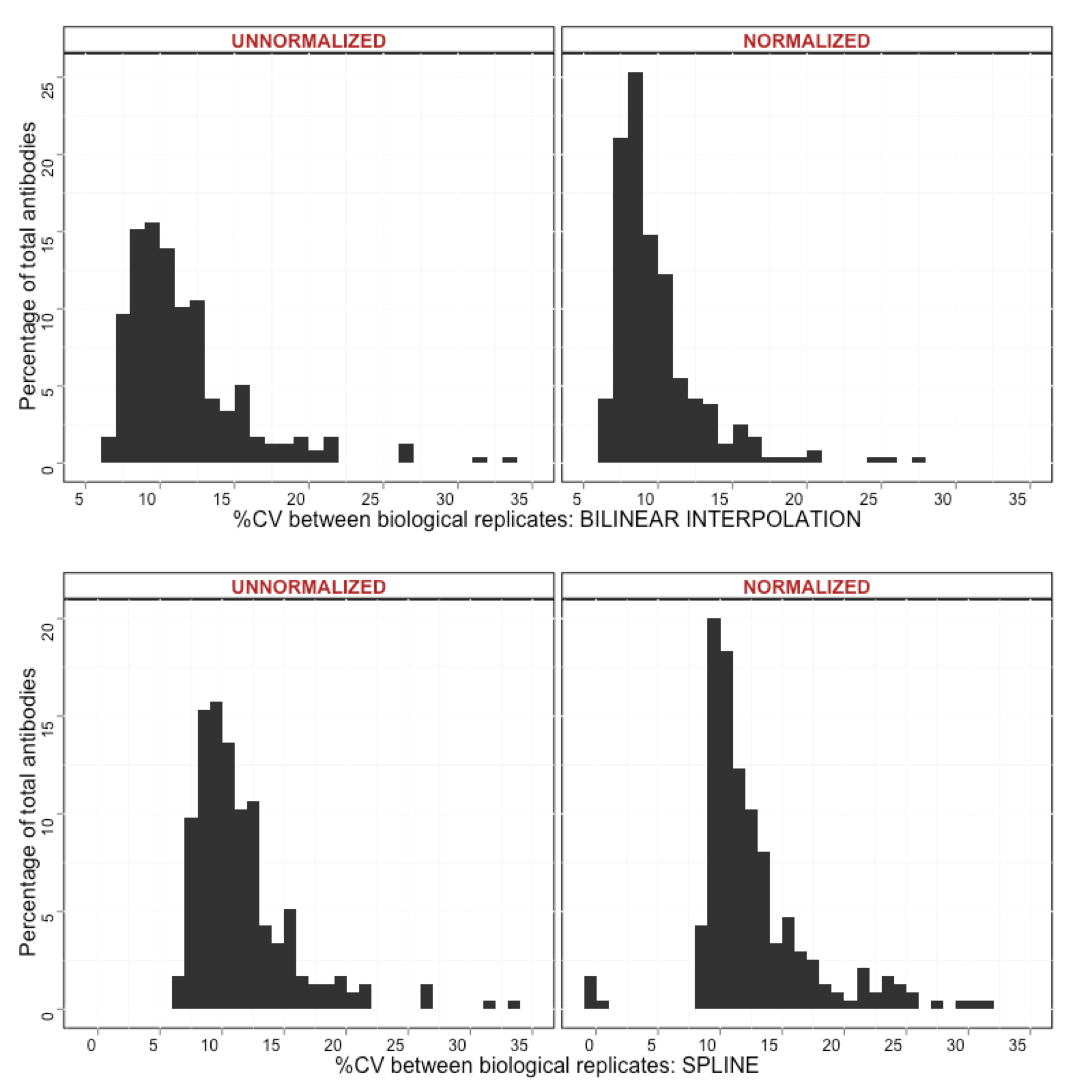

Supplement: Figure S1 — Coefficient of variation between all biological replicates, and across 237 antibody slides used in a melanoma study, before and after normalization of sample intensities using bilinear interpolation and cubic spline. Both methods result in greater agreement between replicates due to normalization. (TIFF) [file pone.0097213.s001.tiff]

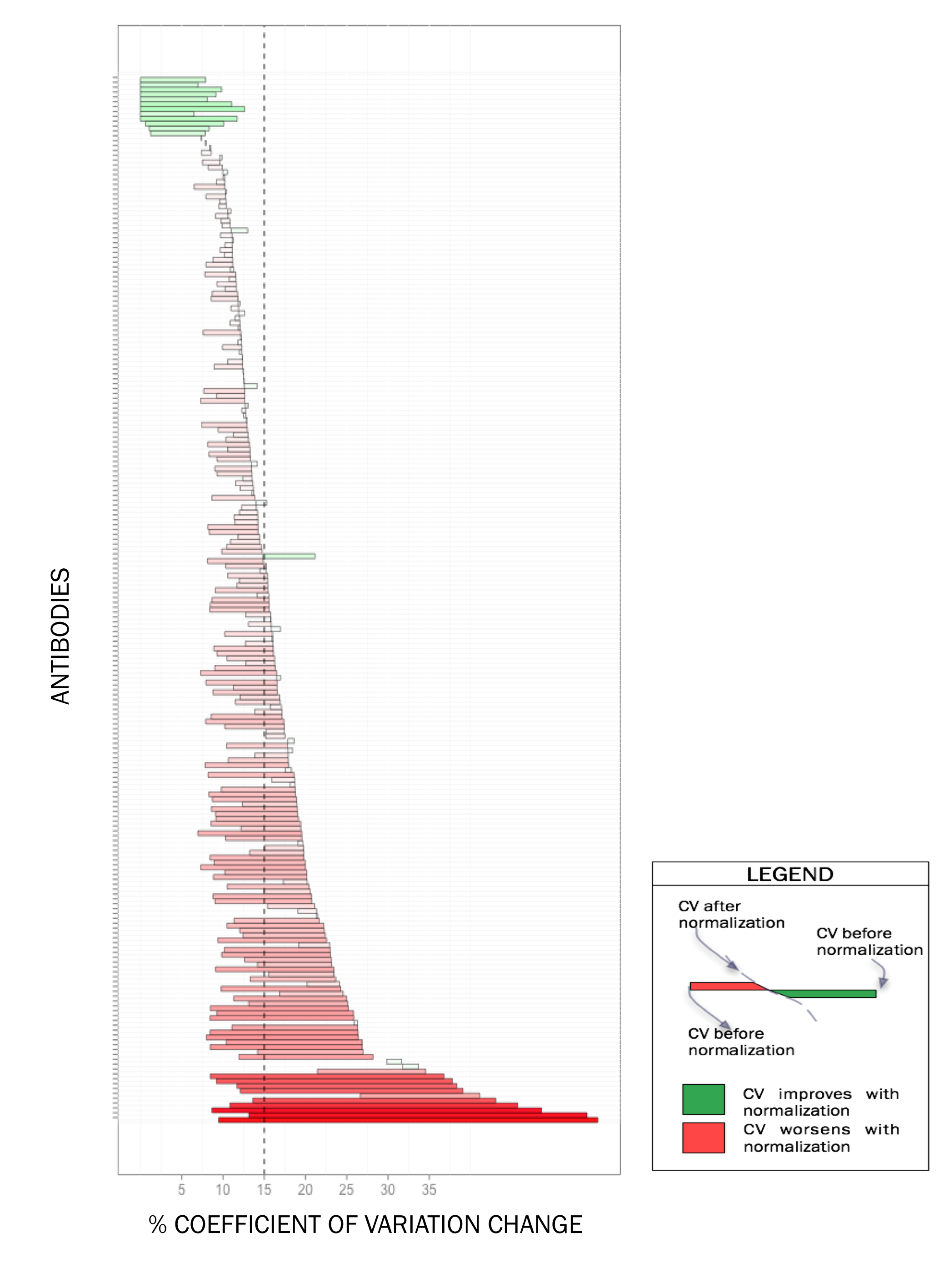

Supplement: Figure S2 — Coefficient of variation between biological replicates in the melanoma study (SET B) appears to worsen for many antibodies when normalization is implemented using the method of Neeley et al. (TIFF) [file pone.0097213.s002.tiff]

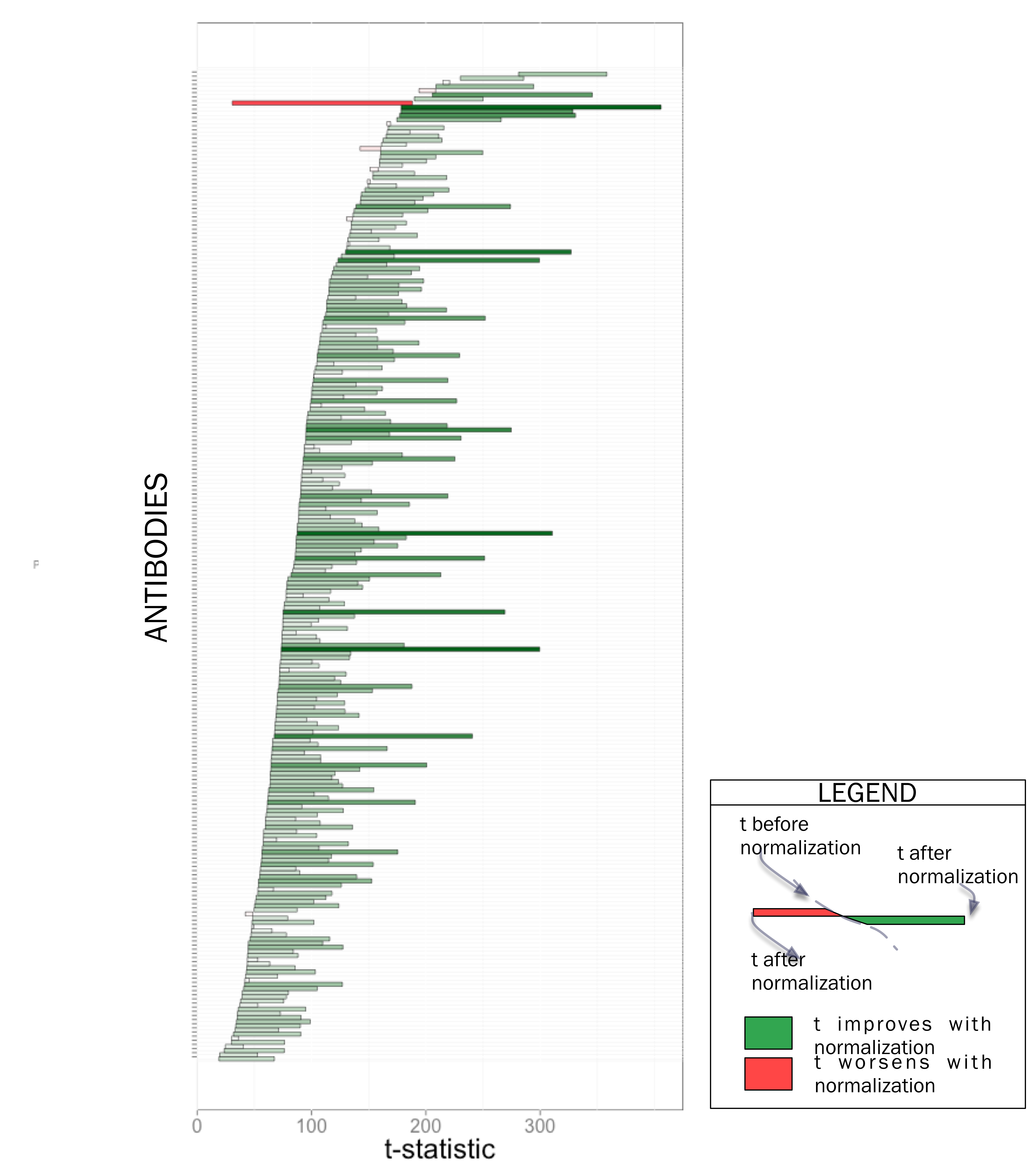

Supplement: Figure S4 — Spatial normalization increases the observed differences between the positive and negative controls in a set of slides (Set B). 229 out of 238 slides (96%) of this set show a clearer separation between the controls after normalization. (TIFF) [file pone.0097213.s004.tiff]
