# Supplementary material for: Spatial Normalization of Reverse Phase Protein Array Data
Source: PLoS One. 2014 Dec 12;9(12):e97213. doi: 10.1371/journal.pone.0097213 (PMC4264691; doi:10.1371/journal.pone.0097213)
Supplement: File S1 — Contains details of supplementary performance assessment of the method. (DOCX) [file pone.0097213.s010.docx]

**Supplemental performance assessment**

To further demonstrate the utility of spatial normalization, we evaluate a statistical parameter known as the Z’-factor [[1](#_ENREF_1)] of each antibody slide before and after normalization. The results obtained from these calculations are consistent with our previous results that spatial normalization improves the signal to noise ratio of measured data. Additionally they demonstrate the different degrees to which normalization may be useful in different contexts.

The Z’-factor is a statistical parameter for assay quality, frequently used to assess the reliability and quality of high throughput studies, and is a dimensionless number equal to the ratio (S/D) of the separation (S) between positive and negative controls to the dynamic range (D) of the assay.

$$Z'=\frac{S}{D}=\frac{(\mu_{pc}-\mu_{nc})-(\sigma_{pc}-\sigma_{nc})}{(\mu_{pc}-\mu_{nc})}$$

Which upon rearranging, reduces to

$$Z'=1-\frac{3\sigma_{pc}+3\sigma_{nc}}{(\mu_{pc}-\mu_{nc})}$$

An ideal assay with invariant signal for positive and negative controls has a separation band equal to the assay’s dynamic range and hence a Z’-factor of 1. As noise increases, Z’-factor drops towards 0. The greater the Z’-factor, the greater the fraction of an assays signal window within which results may be unambiguously attributed to a real effect in the samples under investigation. We chose to use this parameter to further investigate the performance of our method because

1. It is not biased by sample related effects and assesses assay quality based purely on the behavior of technical controls known to be composed of the same chemical material/buffers.
2. It does not require any additional user supplied parameters such as spatial weights or definitions of spot neighborhood
3. It is applicable to any high throughput assay

Our normalization method uses the median dilution in each positive control dilution series against which to normalize the remaining controls and experimental samples. We evaluated Z’-factor using the two dilutions of positive control immediately adjacent to the median control, both before and after normalization. We did not evaluate Z’-factor for the highest and lowest dilution of the positive controls as these dilutions could be influenced by other sources of error such as background noise and signal saturation effects. In both cases, the Z’-factors fell into 3 categories:

Z’ >0.5 ====> an acceptably wide separation band

0.25<Z’<0.5 ====> an assay with small separation band and limited utility

Z’<0.25 ====> an assay in which the signal from positive and negative controls overlap in intensity, hence quantifying signal is not feasible.

We compared the average change in the Z’-Factor of the two dilutions of positive controls immediately adjacent to the median control used for normalization, before and after normalization. We selected these two dilutions as they are likely to be in the linear range of the assay and hence affected more by spatial noise before normalization rather than background and saturation effects. We observed considerable increases in the Z’-Factor, summarized in the table below and in Figure S3.

| Z’-FACTOR | # ANTIBODIES BEFORE NORMALIZATION | # ANTIBODIES AFTER NORMALIZATION |
| --- | --- | --- |
| Z’<0.25 | 37 | 11 |
| 0.25<Z’<0.5 | 63 | 22 |
| Z’ >0.5 | 138 | 205 |

We also evaluated the Welch’s t-statistic to measure the difference between each of these dilutions of positive controls and the set of negative controls. Consistent with our previous observations, we observed increases in this statistic in nearly all measured samples (Figure S4).
